# Serum α-Klotho associated with oral health among a nationally representative sample of US adults

**DOI:** 10.3389/fendo.2022.970575

**Published:** 2022-09-20

**Authors:** Guo-Qiang Chen, Yao Duan, Jin-Feng Wang, Ying Lian, Xiu-Li Yin

**Affiliations:** ^1^ Department of Health Management & Engineering Laboratory for Health Management, The First Affiliated Hospital of Shandong First Medical University & Shandong Provincial Qianfoshan Hospital, Jinan, China; ^2^ Department of Medical Record Management and Statistics, Shandong Provincial Qianfoshan Hospital & The First Affiliated Hospital of Shandong First Medical University, Jinan, China; ^3^ Department of Nursing, Center for Mental Health of Jinan City, Jinan, China; ^4^ Department of Gastroenterology, Shandong Rongjun General Hospital, Jinan, China

**Keywords:** klotho, oral health, periodontitis, tooth loss, dose-response relationship

## Abstract

**Background:**

Low klotho is associated with aging-related traits. However, no study has assessed the association between klotho and oral health in a large sample of population. This study aimed to explore the association between serum α-klotho and oral health in US Adults.

**Methods:**

Data were from the National Health and Nutrition Examination Survey. Oral health parameters included periodontitis, self-rated oral health, and tooth loss. Logistic regression and restricted cubic spline models were adopted to evaluate the associations.

**Results:**

A total of 6187 participants were included in the study. The median of the α-klotho level was 815.2 pg/mL. Serum α-Klotho was significantly lower in participants with poor oral health (all *P <*0.01). Compared with the highest tertile, the lowest tertile of α-klotho was associated with moderate/severe periodontitis, poor-rated oral health, and tooth loss, with OR (95% CI) being 1.21 (1.01, 1.48), 1.26 (1.01, 1.56) and 1.38 (1.05, 1.84), respectively. An increment of per 1 standard deviation in the α-klotho concentration was associated with lower odds of moderate/severe periodontitis (OR: 0.93; 95% CI: 0.87, 0.99). Linear dose-response relationships were found between α-klotho and the odds of moderate/severe periodontitis (*P* for non-linearity=0.88) and poor-rated oral health (*P* for non-linearity=0.66). An L-shaped dose-response relationship was found between levels of α-klotho and the odds of tooth loss (*P* for non-linearity=0.04).

**Conclusions:**

Serum α-klotho was associated with oral health. Further studies are necessary to clarify the potential mechanisms and demonstrate the predictive ability of klotho in oral diseases.

## Introduction

Oral disease has become a serious public health issue worldwide (1, 2). The Global Burden of Disease Study in 2017 has shown that nearly 3.47 billion global people suffered from oral disorders (3), which were more common in socio-economically disadvantaged individuals (4). Oral health, enabling natural eating, speaking, smiling, and socializing, plays a vital role in overall health (5, 6). Deterioration of oral health can lead to various adverse health outcomes, such as cardiovascular diseases (7), cognitive impairment (8), disability (9), and all-cause mortality (10). However, studies have suggested that dental diseases could be prevented and reversed (11, 12). Cohort studies have also suggested that the improvement of oral health might decrease the risk of multiple diseases, including dyslipidemia (13), stroke (14), gastrointestinal cancer (15), new-onset diabetes (16), atrial fibrillation and heart failure (17), and chronic kidney disease (18). Considering the importance of oral health, it is imperative to identify the potential predictive markers and risk factors to improve risk assessment and allow targeted prevention of oral disorders in the early stage.

Klotho protein, a multifunctional protein with antiaging properties, is predominantly expressed in the kidney, parathyroid glands, and choroid plexus of the brain. Secreted klotho is involved in a variety of physiological processes through various mechanisms, such as regulation of serum levels of phosphate and vitamin D, acceleration of oxidative stress (19, 20), etc. Animal models have suggested that mice with lower expression of klotho had shortened lifespan (21), increased inflammatory response, degenerated cognition (22), and delayed skeletal muscle regeneration (23). Epidemiological evidence has suggested the role of low circulating klotho in the risk of various chronic diseases and aging-related traits, including metabolic disorders (19, 24), diminished ovarian reserve (25), and poor physical performance (26, 27). Moreover, klotho was proved to be associated with cerebral small vessel disease (28) and be a biomarker of good functional outcome in patients with acute ischemic stroke (29). These findings have provided insight into the implication of circulating klotho in multiple diseases, that klotho deficiency may not only serve as a potential biomarker but also be a risk factor for developing diseases. Given that oral health could deteriorate with senescence (30, 31), it is crucial to determine the association between serum klotho and oral health, to provide a guide for oral condition prevention. Although abnormal histology and morphology of oral structures were observed in both human (32, 33) and animal specimens (34, 35), it is still unclear whether klotho is associated with oral health in a large population-based study.

Thus, we used data from the National Health and Nutrition Examination Surveys (NHANES) to examine the association between serum α-klotho and oral health and further explore the strength of association in stratified analyses across related characteristics among a nationally representative sample of adults from the United States.

### Null hypotheses:

1. There are non-significant associations of low serum α-klotho with moderate/severe periodontitis, poor-rated oral health, and tooth loss.

2. The dose-responses relationships of serum α-klotho with the risk of moderate/severe periodontitis, poor-rated oral health, and tooth loss are linear.

## Methods

### Sample population

The NHANES is a national program to assess the health and nutritional status of the US sample. A multistage and stratified sampling method was used to identify the participants with good representativeness. Recruited participants were invited to complete standardized questionnaires and undergo a physical examination by laboratory testing after providing written informed consents. Socio-demographic characteristics, physical examinations (including height, weight, blood pressure, clinical periodontal attachment loss, probing pocket depths, and the assessments of remaining tooth), dietary information, and laboratory data were collected. The study protocol was approved by the National Center for Health Statistics Research Ethics Review Board (Protocol #2005-06 and Protocol #2011-17).

**Figure 1:**
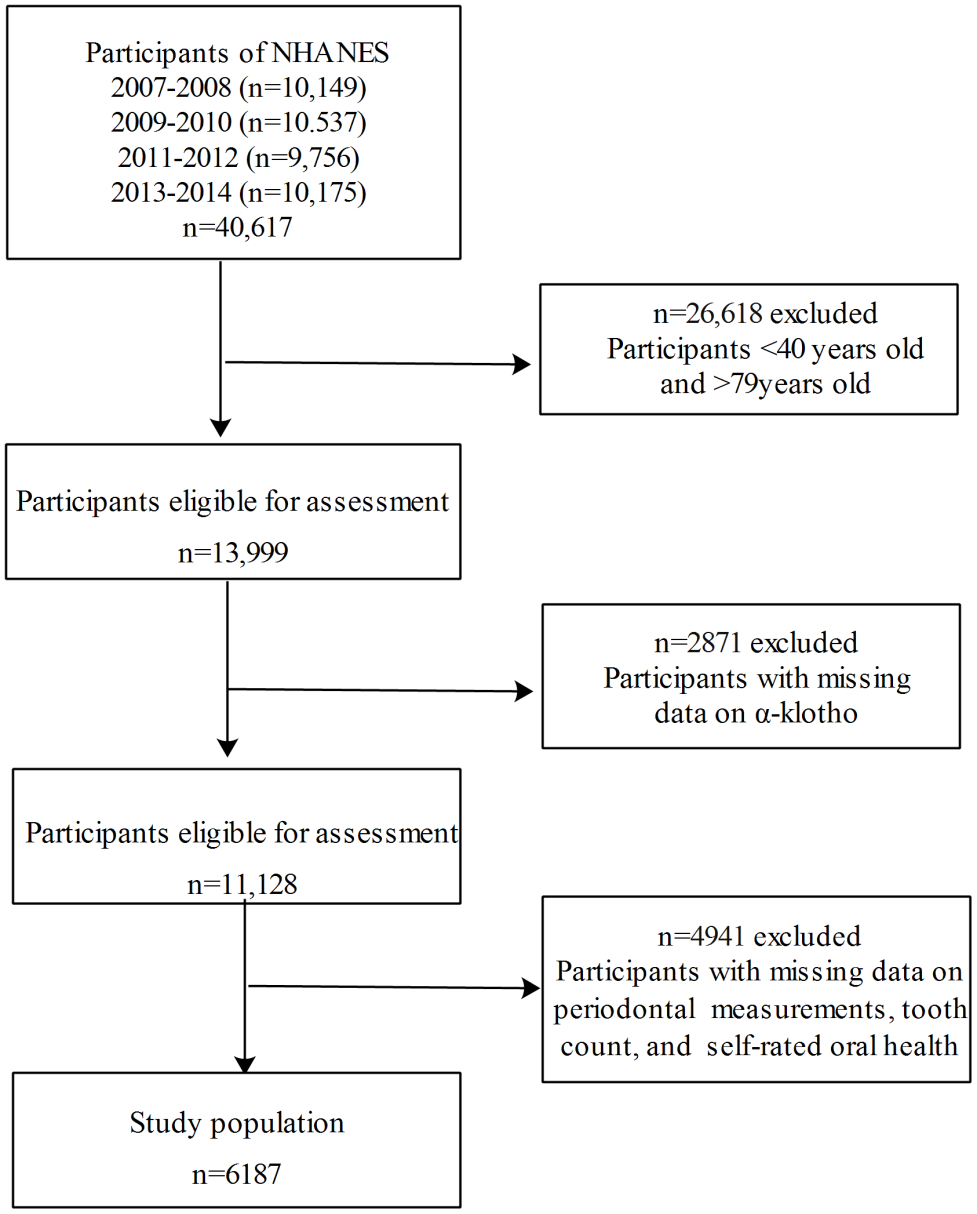
The inclusion and exclusion flowchart of the study population.

Data were combined across 4 continuous NHANES cycles: 2007-2008, 2009-2010, 2011-2012, 2013-2014. Stepwisely excluded those aged <40 or >79 years (n=26618), those with missing data on α-klotho (n=2871), and those with missing data on oral measures (including periodontal examination, self-rated oral health investigation, and assessment of remaining teeth; n=4941), a total of 6187 participants aged 40-79 years with complete information on α-klotho and oral measures were included in the final analysis. The inclusion and exclusion flowchart of the study population is presented in **Figure 1**.

### Serum α-Klotho concentrations

Blood samples were stored at -80^°^C and serum α-klotho was measured among participants aged from 40 to 79 years old. Strictly following the manufacturer’s protocol, serum α-klotho was assayed using a solid-phase sandwich enzyme-linked immunosorbent assay (ELISA 27998, IBL International, Japan). This assay is reported to have a sensitivity of 6 pg/mL. All sample analyses were performed twice and the average of the two values was calculated as the final value of serum α-klotho. More details regarding the laboratory methodology of α-klotho concentrations determination can be available on the NHANES website. In our analyses, α-klotho was categorized by tertiles.

### Assessment of oral health

Oral measures included periodontal examination, investigation of self-rated oral health, and assessment of remaining teeth. A full-mouth periodontal examination, including clinical attachment loss and probing pocket depths, was carried out at six sites per tooth. The periodontal disease diagnosis and staging were carried out following the Center for Disease Control and Prevention and Prevention-American Academy of Periodontology consensus for epidemiologic studies recommendation (36). Periodontitis was categorized as moderate/severe and non/mild periodontitis. Self-reported oral health was assessed *via* responses to the single item: “how would you rate the health of your teeth and gums?” The participants chose one of the following responses: “excellent”, “very good”, “good”, “fair”, or “poor”. Self-rated oral health was categorized as good-rated oral health (excellent/very good/good), and poor-rated oral health (fair/poor). The number of teeth was categorized as a 2-level category, and tooth loss was defined as the number of teeth remaining <21 based on the literature review (37).

### Covariate information

Covariates concerning social demography, lifestyle, and health status adjusted in models were collected, including sex, age, race, educational level, marital status, family poverty income ratio, smoking status, drinking status, physical activity level, body mass index (BMI), diabetes mellitus, and hypertension. Educational level was categorized into two groups (below high school, high school or above). Marital status was categorized into two groups (married/living with a partner, widowed/divorced/separated/never married). Drinking status was defined as <12 or ≥12 alcohol drinks per year (36). Physical activity was asses by the Global Physical Activity Questionnaire (GPAQ) subdivided as high and low/moderate levels. Diabetes mellitus was defined by self-reported physician diagnosis or glucose measures (fasting blood glucose ≥126 mg/dL or glycated hemoglobin ≥6.5%) or current use of glucose-lowering medications. Hypertension was defined as having clinically diagnosed hypertension and/or systolic blood pressure ≥140 mmHg and/or diastolic blood pressure≥90 mmHg. To evaluate the kidney function of the participants, the estimated glomerular filtration rate (eGFR) was estimated using the Chronic Kidney Disease Epidemiology Collaboration equation (38).

### Statistical analyses

The differences in sample characteristics across the tertiles of α-klotho were compared using ANOVA for continuous variables and chi-square tests for categorical variables, respectively. Logistic regression models were used to estimate the odds ratios (ORs) and 95% corresponding confidence intervals (CIs) for oral health parameters. α-Klotho was examined as both a continuous variable and a categorized variable (the highest tertile group was used as the reference group). Model 1 initially adjusted for age and sex. Model 2 adjusted for race, educational level, marital status, family poverty income ratio, smoking status, drinking status, physical activity level, BMI, diabetes mellitus, hypertension, and eGFR, additionally. We also conducted stratified analyses by potential factors, including sex, age (<60 and ≥60 years), BMI (underweight/normal: <24.9 kg/m^2^ and overweight/obese: ≥24.9 kg/m^2^), physical activity (high or low/moderate), the status of hypertension (yes or no), and the status of diabetes mellitus (yes or no). In addition, the shape of the dose-response relationships was explored using restricted cubic splines (RCS) with three knots at the 25th, 50th, and 75th percentiles. All statistical analyses were performed using Stata v14.0.

## Results

### Sample characteristics

**Figure 2:**
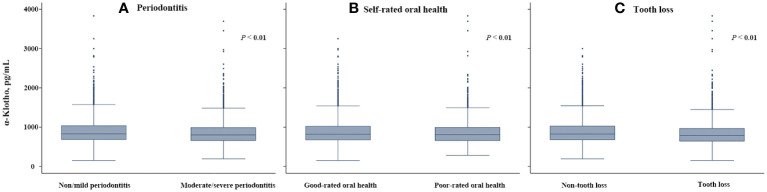
Serum α-klotho levels by oral health status compared by Mann-Whitney U tests: **(A)** Moderate/severe periodontitis, **(B)** Poor-rated oral health, and **(C)** Tooth loss.

The characteristics of the study participants are shown in **Table 1**. Of 6178 participants, the prevalence of moderate/severe periodontitis, poor-rated oral health, and tooth loss were 54.84%, 34.88%, and 27.62%, respectively, and the median serum α-klotho level was 815.2 pg/mL (25th-75th percentiles: 665.9-1010.7 pg/mL). Participants in the lowest tertile group (<716.3 pg/mL) were older, more likely to be male and had lower eGFR, higher BMI and higher prevalence of hypertension. The prevalence of moderate/severe periodontitis (58.53%), poor-rated oral health (36.58%), and tooth loss (31.20%) were the highest in the lowest tertile group. **Figure 2** shows the serum α-klotho level by oral health status. α-Klotho levels were significantly lower in participants with poorer oral health, with the medians being 800.5, 804.7, and 790.4 pg/mL for moderate/severe periodontitis, poor-rated oral health, and tooth loss, respectively (all *P*<0.01).

**Table 1:** Sample characteristics.

Characteristics	Overall	Tertile 1 of α-klotho	Tertile 2 of α-klotho	Tertile 3 of α-klotho	*P*-value
Age (years)	56.09 (10.67)	57.19 (10.73)	56.08 (10.42)	55.01 (10.15)	<0.01
Sex (%)					<0.01
Male	3026 (48.91)	1068 (51.74)	1048 (50.85)	910 (44.13)	
Female	3161 (51.09)	996 (48.26)	1013 (49.15)	1152 (55.87)	
Race (%)					<0.01
Non-Hispanic White	2652 (42.86)	938 (45.45)	942 (45.71)	772 (37.45)	
Non-Hispanic Black	1243 (20.09)	399 (19.33)	345 (16.74)	499 (24.20)	
Mexican-American	949 (15.34)	313 (15.16)	339 (16.45)	297 (14.40)	
Other Hispanic	645 (10.43)	207 (10.03)	195 (9.46)	243 (11.78)	
Other race	698 (11.28)	207 (10.03)	240 (11.64)	251 (12.17)	
Educational level (%)					<0.01
Below high school	1509 (24.39)	535 (25.92)	507 (24.60)	467 (22.65)	
High school and above	4678 (75.61)	1529 (74.08)	1554 (75.40)	1595 (77.35)	
Marriage status (%)					0.12
Married/Living with partner	4089 (66.08)	1374 (66.55)	1378 (66.88)	1337 (64.82)	
Widowed/Divorced/Separated/Never married	2098 (33.92)	690 (33.45)	683 (33.12)	725 (35.18)	
Family poverty income ratio (%)					0.82
≤1	1111 (17.95)	379 (18.35)	358 (17.38)	374 (18.14)	
1-1.84	1352 (21.85)	463 (22.45)	445 (21.59)	444 (21.53)	
≥1.85	3724 (60.20)	1222 (59.20)	1258 (61.03)	1245 (60.33)	
Smoking status (%)					<0.01
Never	3384 (54.70)	1028 (49.81)	1109 (53.81)	1247 (60.48)	
Current	1091 (17.63)	411 (19.91)	362 (17.56)	318 (15.42)	
Former	1712 (27.67)	625 (30.28)	590 (28.63)	497 (24.10)	
Drinking status (%)					<0.01
<12 alcohol drinks per year	4543 (73.46)	1582 (76.64)	1533 (74.39)	1428 (69.25)	
≥12 alcohol drinks per year	1644 (26.54)	482 (23.36)	528 (25.61)	624 (30.75)	
Physical activity level (%)					0.14
High/Moderate	2976 (48.10)	969 (46.96)	1018 (49.39)	989 (47.96)	
Low	3211 (51.90)	1095 (53.04)	1043 (50.61)	1073 (52.04)	
Hypertension (%)	1363 (22.03)	510 (24.72)	431 (20.89)	422 (20.47)	<0.01
Diabetes mellitus (%)	1308 (21.14)	446 (21.62)	401 (19.46)	461 (22.35)	0.06
BMI (kg/m2)	29.54 (6.49)	29.84 (6.23)	29.47 (6.51)	29.32 (6.72)	<0.01
eGFR (mL/min/1.73 m²)	87.83 (19.19)	84.50 (21.21)	88.34 (18.18)	90.65 (17.49)	<0.01
Moderate/severe periodontitis (%)	3393 (54.84)	1208 (58.53)	1131 (54.88)	1054 (51.12)	<0.01
Poor-rated oral health (%)	2158 (34.88)	755 (36.58)	723 (35.08)	680 (32.98)	0.05
Tooth loss (%)	1709 (27.62)	644 (31.20)	569 (27.61)	496 (24.05)	<0.01

Continuous variables are presented as means (standard deviations); Categorical variables are presented as frequencies (percentages).

Tertile 1, <716.3 pg/mL; Tertile 2, 716.3-931.8 pg/mL; Tertile 3, >931.8 pg/mL; BMI, body mass index; eGFR, estimated glomerular filtration rate.

### Associations of α-klotho with oral health parameters

The associations of α-klotho with oral health parameters are presented in **Table 2**. Compared with the highest tertile, the lowest tertile of α-klotho was associated with moderate/severe periodontitis, poor-rated oral health, and tooth loss, with OR (95% CI) being 1.21 (1.01, 1.48), 1.26 (1.01, 1.56) and 1.38 (1.05, 1.84), respectively, after adjusting for potential confounders. The trend test also showed that with the decrease of α-klotho, the odds of tooth loss increased (*P* for trend =0.01). An increment of per 1 standard deviation in α-klotho was associated with lower odds of moderate/severe periodontitis (OR: 0.93; 95% CI: 0.87, 0.99).

**Table 2:** Weighted ORs (95%CIs) of the association between α-klotho and oral health.

Outcome	Model 1	Model 2
Moderate/severe periodontitis		
Tertile 1 of α-klotho	1.31 (1.10, 1.56)	1.21 (1.01, 1.48)
Tertile 2 of α-klotho	1.09 (0.92, 1.30)	1.10 (0.92, 1.33)
Tertile 3 of α-klotho	Ref.	Ref.
* P* for trend	0.11	0.17
Per 1-SD increase of α-klotho	0.90 (0.85, 0.96)	0.93 (0.87, 0.99)
Poor-rated oral health		
Tertile 1 of α-klotho	1.30 (1.09, 1.55)	1.26 (1.01, 1.56)
Tertile 2 of α-klotho	1.17 (0.99, 1.38)	1.17 (0.96, 1.43)
Tertile 3 of α-klotho	Ref.	Ref.
* P* for trend	0.03	0.09
Per 1-SD increase of α-klotho	0.91 (0.83, 1.00)	0.92 (0.83, 1.02)
Tooth loss		
Tertile 1 of α-klotho	1.44 (1.13, 1.83)	1.38 (1.05, 1.84)
Tertile 2 of α-klotho	1.21 (1.01, 1.47)	1.32 (1.04, 1.69)
Tertile 3 of α-klotho	Ref.	Ref.
* P* for trend	0.02	0.01
Per 1-SD increase of α-klotho	0.91 (0.80, 1.01)	0.92 (0.80, 1.05)

Data are presented as ORs (95%CIs).

Model 1: Adjusted for age and sex.

Model 2: Adjusted for Model 1 covariates plus race, educational level, marital status, family poverty income ratio, smoking status, drinking status, physical activity level, BMI, diabetes mellitus, hypertension, and eGFR.

Tertile 1, <716.3 pg/mL; Tertile 2, 716.3-931.8 pg/mL; Tertile 3, >931.8 pg/mL;OR, odds ratio; 95% CI, 95% confidence interval; Ref, reference group; BMI, body mass index; SD, standard deviation; eGFR, estimated glomerular filtration rate.

### Dose-response relationship of serum α-klotho level with oral health

**Figure 3:**
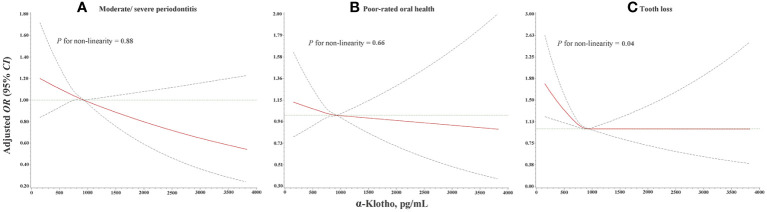
Dose-response relationship of α-klotho with **(A)** moderate/severe periodontitis, **(B)** poor-rated oral health, and **(C)** tooth loss from RCS analysis with three knots at the 25th, 50th, and 75th percentiles with adjustment for age, sex, race, educational level, marital status, family poverty income ratio, smoking status, drinking status, physical activity level, BMI, diabetes mellitus, hypertension, and eGFR. OR, odds ratio; 95% CI, 95% confidence interval; RCS, restricted cubic splines; BMI, body mass index; eGFR, estimated glomerular filtration rate.

The dose-response relationship of serum α-klotho level with oral health is presented in **Figure 3**. Linear dose-response relationships were found between serum α-klotho and the odds of moderate/severe periodontitis (*P* for non-linearity =0.88) and poor-rated oral health (*P* for non-linearity =0.66). An L-shaped dose-response relationship was found between α-klotho and the odds of tooth loss (*P* for non-linearity =0.04), suggesting that under the highest tertile of α-klotho concentration, the odds of tooth loss increase with the decline of the serum α-klotho concentration.

### Stratified analysis of the association between α-klotho and oral health

The stratified analysis of the association between α-klotho and oral health is shown in **Table 3**. Compared with the highest tertile, the lowest tertile of α-klotho was associated with poor-rated oral health and tooth loss in females (OR: 1.46, 95% CI: 1.10, 1.93 for poor-rated oral health; OR: 1.57, 95% CI: 1.16, 2.13 for tooth loss), adults with low physical activity (OR: 1.53, 95% CI: 1.16, 2.02 for poor-rated oral health; OR: 1.49, 95% CI: 1.05, 2.10 for tooth loss), and adults who were overweight or obese (OR: 1.38, 95% CI: 1.10, 1.72 for poor-rated oral health; OR: 1.55, 95% CI: 1.10, 2.16 for tooth loss).

**Table 3:** Weighted ORs (95%CIs) of the association between α**-**klotho and oral health in stratified analyses.

Characteristics	Moderate/severe periodontitis				Poor-rated oral health				Tooth loss			
	Tertile 3	Tertile 2	Tertile 1	*P* for trend	Tertile 3	Tertile 2	Tertile 1	*P* for trend	Tertile 3	Tertile 2	Tertile 1	*P* for trend
Sex												
Female	Ref.	1.24 (0.98, 1.58)	1.19 (0.94, 1.51)	0.24	Ref.	1.04 (0.79, 1.38)	1.46 (1.10, 1.93)	0.07	Ref.	1.60 (1.17, 2.19)	1.57 (1.16, 2.13)	<0.01
Male	Ref.	1.10 (0.83, 1.47)	1.13 (0.85, 1.51)	0.43	Ref.	1.11 (0.80, 1.55)	1.15 (0.84, 1.59)	0.14	Ref.	1.22 (0.82, 1.83)	1.30 (0.88, 1.92)	0.43
Age												
<60 years	Ref.	1.24 (0.99, 1.55)	1.32 (1.06, 1.64)	0.01	Ref.	1.24 (0.98, 1.57)	1.25 (0.99, 1.58)	0.12	Ref.	1.45 (1.02, 2.05)	1.39 (0.91, 2.11)	0.11
≥60 years	Ref.	1.03 (0.69, 1.55)	0.99 (0.78, 1.25)	0.56	Ref.	0.93 (0.65, 1.34)	1.20 (0.85, 1.69)	0.78	Ref.	1.26 (0.88, 1.80)	1.56 (1.15, 2.12)	0.01
Physical activity												
High/Moderate	Ref.	1.12 (0.85, 1.47)	1.16 (0.86, 1.57)	0.29	Ref.	1.05 (0.74, 1.50)	1.07 (0.72, 1.60)	0.78	Ref.	1.28 (0.89, 1.84)	1.29 (0.87, 1.92)	0.17
Low	Ref.	1.13 (0.89, 1.45)	1.21 (0.89, 1.62)	0.24	Ref.	1.33 (1.01, 1.74)	1.53 (1.16, 2.02)	<0.01	Ref.	1.38 (0.97, 1.98)	1.49 (1.05, 2.10)	0.01
BMI												
Underweight/Normal	Ref.	1.09 (0.76, 1.56)	1.00 (0.69, 1.43)	0.96	Ref.	0.98 (0.69, 1.38)	1.09 (0.74, 1.60)	0.72	Ref.	0.89 (0.53, 1.48)	0.95 (0.59, 1.54)	0.81
Overweight/Obese	Ref.	1.24 (0.97, 1.59)	1.24 (0.99, 1.56)	0.10	Ref.	1.22 (0.99, 1.51)	1.38 (1.10, 1.72)	0.03	Ref.	1.38 (1.04, 1.84)	1.55 (1.10, 2.16)	0.02
Hypertension												
No	Ref.	1.19 (0.95, 1.50)	1.17 (0.94, 1.47)	0.23	Ref.	1.06 (0.86, 1.30)	1.20 (0.96, 1.52)	0.29	Ref.	1.25 (0.94, 1.67)	1.38 (1.00, 1.91)	0.06
Yes	Ref.	0.89 (0.60, 1.30)	1.17 (0.75, 1.81)	0.78	Ref.	1.45 (0.93, 2.27)	1.49 (0.98, 2.24)	0.08	Ref.	1.85 (1.01, 3.42)	1.69 (1.03, 2.78)	0.04
Diabetes mellitus												
No	Ref.	1.15 (0.93, 1.42)	1.28 (1.04, 1.57)	0.04	Ref.	1.20 (0.97, 1.49)	1.27 (0.98, 1.65)	0.15	Ref.	1.27 (0.95, 1.68)	1.31 (0.94, 1.81)	0.13
Yes	Ref.	1.08 (0.68, 1.70)	0.92 (0.60, 1.42)	0.55	Ref.	1.22 (0.69, 2.15)	1.28 (0.93, 1.75)	0.28	Ref.	1.38 (0.90, 2.10)	1.45 (0.94, 2.24)	0.07

Data are presented as ORs (95%CIs).

Adjusted for age, sex, race, educational level, marital status, family poverty income ratio, smoking status, drinking status, physical activity level, BMI, diabetes mellitus, hypertension, and eGFR.

Tertile 1, the lowest; Tertile 2, the median; Tertile 3, the highest; OR, odds ratio; 95% CI, 95% confidence interval; Ref, reference group; BMI, body mass index; eGFR, estimated glomerular filtration rate.

## Discussion

In the present study, we explored the association between α-klotho and oral health among a nationally representative sample of U.S. adults. The levels of α-klotho were significantly lower in participants with poorer oral health than those in their counterparts. Meanwhile, low α-klotho was associated with increased odds of moderate/severe periodontitis, poor-rated oral health, and tooth loss. And, the associations between α-klotho and oral health parameters were presented in dose-response manners. Furthermore, stratified analyses demonstrated the association of α-klotho with oral health across diverse subgroups.

The observed association between klotho and oral health in our study was consistent with previous studies. A cross-sectional study conducted in China showed that compared with those in the healthy counterparts, the levels of klotho in the gingival crevicular fluid and gingival tissues of chronic periodontitis patients were significantly lower (32). Another cross-sectional study conducted in the US demonstrated that increased tooth pulp density was observed in a hyperphosphatemic familial tumoral calcinosis patient caused by mutations in klotho (33). Moreover, a meta-analysis including 18 studies conducted in 2018 showed that lower klotho was associated with oral malignancy (OR: 0.034; 95% CI: 0.002, 0.630), indicating its function as a biomarker in oral cancer (39). Similar findings were also found in animal models. A study conducted in Japan showed that compared with the phenotype of wild-type, abnormal histology of the periodontal tissue caused by the disrupted signal of αklotho/fibroblast growth factor (FGF) 23 pathway, including narrowed periodontal spaces, amorphous structures in the periodontal ligament, and irregular distribution of periodontal fibers, was observed in klotho-deficient mice (34). Likewise, another study demonstrated that klotho-deficient mice presented abnormal distribution and morphology of odontoblasts and dentin matrix of the mandibular incisors, meanwhile, the apoptosis reaction was detected in the embedded odontoblast-like cells and pulpal cells only among klotho-deficient mice, compared with wild-type counterparts (35). Our study extended existing evidence by demonstrating the association in a sample of large population. Notably, an L-shaped relationship was found between α-klotho level and the odds of tooth loss, suggesting that the odds of tooth loss increase with the decline of the serum α-klotho concentration. The observed associations suggested that serum α-klotho levels may be a potential biomarker of oral diseases and deficiency in α-klotho may play a part in their progression, which can expand our understanding of the potential roles of klotho in oral health and provide a novel insight into preventing the development of oral diseases.

Several possible mechanisms may explain the association between klotho and oral health. First, klotho could inhibit oxidative stress and down-regulate cell apoptosis in human periodontal ligament stem cells (hPLSCs) (32, 40), which were considered a potential mechanism underlying oral conditions (41). Previous studies have shown that under a simulated oxidative stress circumstance by H_2_O_2_, the level of reactive oxygen species (ROS) and malondialdehyde were both reduced, meanwhile, the activity of antioxidant enzymes (including superoxide dismutase, glutathione peroxidase, and catalase) was restored, by the treatment of klotho protein in hPLSC. Moreover, the expressions of apoptotic-related proteins (including BAX and Caspase-3) in hPLSCs were also reduced by the addiction of klotho (32, 40). Thus, a deficiency of klotho may attribute to the accumulation of ROS and the acceleration of cell apoptosis, leading to the deterioration of oral health eventually. Second, lack of klotho may induce the abnormal synthesis of alkaline phosphatase (ALP) and bone matrix proteins (including dentin matrix protein-1 [DMP-1] and osteopontin [OPN]) in tooth (34, 35), which are essential for dentin formation and mineralization (42). Animal studies have shown that a weak reaction for ALP immune-staining and irregular distributions of DMP-1 and OPN were observed in oral tissues of klotho-deficient mice (34, 35). Third, α-klotho regulates the metabolism of main mineral components (including calcium [Ca] and phosphorus [P]) of tooth (19). Lower concentrations of Ca and P in incisors of klotho-deficient mice were observed in a previous study (35). Therefore, the reduction of Ca and P in dental tissue by the deficiency of klotho may result in oral conditions. Besides anti-oxidative stress, dentin proteins synthesis, and mineral homeostasis, klotho may also take part in substance metabolism (glucose, fatty acid, and bile acid), responses to feeding and fasting, energy expenditure, hypothalamic–pituitary–adrenal axis activity, and sympathetic activity, underlining its potential endocrine role in various human disorders, including cancers (liver, colon, breast and prostate), bile acid diarrhea, chronic kidney disease, type 2 diabetes mellitus, obesity, nonalcoholic fatty liver disease, cardiovascular disease, anorexia nervosa and tumor-induced osteomalacia (19).

In stratified analyses, we found that low α-klotho was associated with poor-rated oral health and tooth loss only in females. This might be explained by the effect of sex hormone change in menopause among females. A longitudinal study of 1341 postmenopausal women with a mean follow-up of 5.1-year showed that 28.7% of participants lost at least one tooth during follow-up (43). Besides, it is reported that bone loss was at an accelerated rate of 2% per year during menopause and lasted for six years thereafter (44). Of note, osteoporosis was considered a sign of periodontal diseases in postmenopausal women (45). Moreover, this finding was only pronounced in adults with low or moderate physical activity and those who were overweight or obese, compared with their counterparts. The preference for health-compromising behaviors in those two subgroups may explain part of the observed discrepancies, such as a diet rich in sugars (46), consumption of beverages (47, 48), use of tobacco (49), poor oral hygiene (47, 50), suggesting that oral health education programs aimed at establishing favorable health-related behaviors should be implemented among these two groups of people. We also found that the association between low α-klotho and tooth loss was stronger in those with hypertension. One potential reason may be that hypertension is a risk factor for tooth loss (51), indicating a cumulative effect of hypertension and low α-klotho on tooth loss.

Our study examined the association between α-klotho and oral health based on a nationally representative sample, which facilitates the generalization of the findings. Second, the dose-response relationships in our findings help us to evaluate how the odds of oral conditions change with the continuous decrease of α-klotho. However, several limitations of our study should be noted. First, the cross-sectional design does not allow us to infer causation between α-klotho and oral diseases or conditions and to examine whether the klotho level could be corrected to improve oral health. Second, confounding factors, including dental visits, and history of head and neck radiotherapy were not assessed in the present study,which might have influenced our findings. Third, due to the constraints of the NHANES database, the parameters of oral health were not defined comprehensively: 1. Periodontitis was defined by clinical attachment loss and probing pocket depths without consideration of saliva or gingival crevicular fluid in our study. 2. Tooth loss was simply defined by the number of remaining teeth, overlooking the cause of the tooth loss in the present study. Fourth, as shown in **Supplementary Table 1**, Some characteristics between included and excluded participants were different, which might bring about bias in estimating the associations. Thus, prospective studies and randomized controlled trials incorporating a more extensive investigation of oral health markers and other confounding factors are needed to fully clarify the causal relationship.

## Conclusions

In conclusion, we found that serum α-klotho is associated with oral health in US adults. And the association presents in a dose-response manner. It may be of significant public health and clinical importance that provide new insights into the implication of klotho in oral diseases. Further studies are necessary to clarify the potential mechanisms underlying the association and demonstrate the predictive ability of α-klotho in oral diseases.

## Data availability statement

The datasets presented in this article are not readily available because of privacy or ethical restrictions. The data used and analyzed during the present study are available from the corresponding author on reasonable request.

## Ethics statement

The study protocol was approved by the National Center for Health Statistics Research Ethics Review Board. The patients/participants provided their written informed consent to participate in this study.

## Author contributions

X-LY and G-QC conceived and designed the study. G-QC analyzed the data and wrote the manuscript. YL, J-FW and YD provided comments and technical advice. All authors revised the manuscript critically for important intellectual content, gave final approval of the version to be published, and agree to be accountable for all aspects of the work.

## Funding

This work was supported by Shandong Provincial Key Research and Development Program (grant no. 2019GSF108196), Center of China–US Sports Economics and Health Engineering of Shandong (grant no. SDCA20191013), Academic Promotion Programme of Shandong First Medical University (grant no. 2019QL013).

## Acknowledgments

We gratefully acknowledge all of the study participants and staff for their assistance during the study.

## Conflict of interest

The authors declare that the research was conducted in the absence of any commercial or financial relationships that could be construed as a potential conflict of interest.

## Publisher’s note

All claims expressed in this article are solely those of the authors and do not necessarily represent those of their affiliated organizations, or those of the publisher, the editors and the reviewers. Any product that may be evaluated in this article, or claim that may be made by its manufacturer, is not guaranteed or endorsed by the publisher.
