# Supplementary material for: Serum α-Klotho associated with oral health among a nationally representative sample of US adults
Source: Front Endocrinol (Lausanne). 2022 Sep 20;13:970575. doi: 10.3389/fendo.2022.970575 (PMC9530453; doi:10.3389/fendo.2022.970575)
Supplement: Supplementary file 1 [file Table_1.docx]

**Supplementary Table 1│** Characteristics between the included and excluded participants

| **Characteristics** | **Participants included**  **(n=6187)** | **Participants excluded**  **(n=7812)** | ***P*-value** |
| --- | --- | --- | --- |
| Age (years) | 56.09 (10.67) | 55.01 (10.15) | <0.01 |
| Sex (%) |  |  | 0.67 |
| Male | 3026 (48.91) | 3792 (48.54) |  |
| Female | 3161 (51.09) | 4020 (51.46) |  |
| Race (%) |  |  | <0.01 |
| Non-Hispanic White | 2652 (42.86) | 3308 (42.35) |  |
| Non-Hispanic Black | 1243 (20.09) | 1920 (24.58) |  |
| Mexican-American | 949 (15.34) | 1096 (14.03) |  |
| Other Hispanic | 645 (10.43) | 864 (11.06) |  |
| Other race | 698 (11.28) | 624 (7.98) |  |
| Educational level (%) |  |  | <0.01 |
| Below high school | 1509 (24.39) | 2542 (32.54) |  |
| High school and above | 4678 (75.61) | 5270 (67.46) |  |
| Marriage status (%) |  |  | <0.01 |
| Married/Living with partner | 4089 (66.08) | 4713 (60.33) |  |
| Widowed/Divorced/Separated/Never married | 2098 (33.92) | 3099 (39.67) |  |
| Family poverty income ratio (%) |  |  | <0.01 |
| ≤1 | 1111 (17.95) | 1780 (22.78) |  |
| 1-1.84 | 1352 (21.85) | 1916 (24.53) |  |
| ≥1.85 | 3724 (60.20) | 4116 (52.69) |  |
| Smoking status (%) |  |  | <0.01 |
| Never | 3384 (54.70) | 3769 (48.24) |  |
| Current | 1091 (17.63) | 1756 (22.48) |  |
| Former | 1712 (27.67) | 2287 (29.28) |  |
| Drinking status (%) |  |  | <0.01 |
| <12 alcohol drinks per year | 4543 (73.46) | 5334 (68.28) |  |
| ≥12 alcohol drinks per year | 1644 (26.54) | 2478 (31.72) |  |
| Physical activity level (%) |  |  | <0.01 |
| High/Moderate | 2976 (48.10) | 3257 (41.69) |  |
| Low | 3211 (51.90) | 4555 (58.31) |  |
| Hypertension (%) | 1363 (22.03) | 2321 (29.71) | <0.01 |
| Diabetes mellitus (%) | 1308 (21.14) | 2201 (28.17) | <0.01 |
| BMI (kg/m^2^) | 29.54 (6.49) | 29.60 (7.00) | 0.69 |
| eGFR (mL/min/1.73 m²) | 87.83 (19.19) | 85.74 (20.96) | <0.01 |

*Continuous variables are* *presented as means (standard deviations); Categorical variables are presented as frequencies (percentages).*

*BMI: body mass index; eGFR: estimated glomerular filtration rate.*
